# The p53 status of cultured human premalignant oral keratinocytes.

**DOI:** 10.1038/bjc.1994.356

**Published:** 1994-10

**Authors:** J. E. Burns, L. J. Clark, W. A. Yeudall, R. Mitchell, K. Mackenzie, S. E. Chang, E. K. Parkinson

**Affiliations:** CRC Laboratories, Garscube Estate, Bearsden, Glasgow, UK.

## Abstract

**Images:**


© Macmillan Press Ltd., 1994

### The p53 status of cultured human premalignant oral keratinocytes

J.E. Burns[1], L.J. Clark[1], W.A. Yeudall[2+], R. Mitchell[3], K. Mackenzie[4], S.E. Chang[5] & E.K. Parkinson

[1]CRC Laboratories, Garscube Estate, Switchback Road, Bearsden, Glasgow G61 1BD, UK; [2]Department of Oral Medicine, Pathology and Microbiology, University of Bristol, Bristol, UK; [3]Department of Oral and Maxillofacial Surgery, City Hospital, Greenbank Road, Edinburgh EH10 5SB, UK; [4]Department of Otolaryngology, Glasgow Royal Infirmary, Castle St, Glasgow G4 0SF, UK; [5]Department of Dental Sciences, Royal College of Surgeons of England, 35-43 Lincoln's Inn Fields, London WC2A 3PN, UK.

**Summary** Around 60% of oral squamous cell carcinomas (SCCs) have been shown to harbour p53 mutations, and other studies have demonstrated mutant p53 genes in normal and dysplastic squamous epithelium adjacent to these SCCs. In line with these earlier studies we show here that DOK, a keratinocyte cell line derived from a dysplasia, displays elevated levels of p53 protein and harbours a 12 bp in-frame deletion of the p53 gene spanning codons 188-191. In contrast, the coding region of the p53 gene was normal in a series of six benign recurrent laryngeal papillomas and a series of four premalignant oral erythroplakia biopsies and their cell cultures. All but one of these lesions were free of malignancy at the time of biopsy, in contrast to the premalignant lesions studied by previous investigators, but keratinocytes cultured from these lesions all displayed a partially transformed phenotype that was less pronounced than that of DOK. Since three out of four of the erythroplakia patients developed SCC within 1 year of biopsy, these lesions were by definition premalignant. The availability of strains of partially transformed keratinocytes from premalignant erythroplakias which possess normal p53 genes should enable us to test the role of mutant p53 in the progression of erythroplakia to SCC. The premalignant tissues and cultures were also tested for the presence of human papillomavirus (HPV), which is known to inactivate p53 function in some cases. Only the benign papillomas were shown to contain high levels of either HPV 6 or HPV 11 E6 DNA, but not both, and none of the samples contained detectable levels of HPV 16, HPV 18 or HPV 33 E6 DNA or L1 DNA of several other HPV types. There was therefore no evidence to suggest that p53 was being inactivated by a highly oncogenic HPV in these samples.

Squamous cell carcinoma of the head and neck (SCC-HN) is an extremely common tumour worldwide (Pindborg, 1984; Million *et al*., 1989), yet little is known of the molecular mechanisms which result in its development. Some SCC-HN arise from premalignant lesions such as papillomas, leukoplakias and erythroplakias, while others do not. Papillomas in humans are essentially benign, with only a small percentage (less than 1%) progressing to malignancy, but leukoplakias and more usually erythroplakias do progress to malignancy (Pindborg, 1985).

The p53 tumour-suppressor gene has been implicated in the pathogenesis of SCC-HN and is commonly mutated or deleted in tumours from Caucasian populations (Brachman *et al*., 1992; Jung *et al*., 1992; Maestro *et al*., 1992; Somers *et al*., 1992; Boyle *et al*., 1993; Burns *et al*., 1993; Chung *et al*., 1993; Nees *et al*., 1993). In many cases this leads to stabilisation of the p53 protein, rendering it unusually detectable by immunocytochemistry (Field *et al*., 1991; Gusterson *et al*., 1991; Maestro *et al*., 1992; Burns *et al*., 1993). Elevated levels of p53 protein have also been reported in histologically normal oral mucosa and dysplastic tissue adjacent to SCCs of the oral cavity (Gusterson *et al*., 1991; Ogden *et al*., 1992) and larynx (Dolcetti *et al*., 1992) and also in the more basal cells of recurrent (Clark *et al*., 1993*a*) but not solitary (Ogden *et al*., 1992) laryngeal papillomas. This has led some authors to speculate that mutation of p53 might be an early event in the development of SCC-HN (Dolcetti *et al*., 1992), and very recently it has been shown that at least in some instances both histologically normal (Nees *et al*., 1993) and dysplastic (Boyle *et al*., 1993) oral epithelia harbour mutant p53 genes. Most of these mutants, however, are likely to be of the stable (Oren *et al*., 1981) and possibly gain-of-function type (Halevy *et al*., 1990) since they result in increased levels of p53 protein (Dolcetti *et al*., 1992; Nees *et al*., 1993). It is less certain whether loss of p53 suppressor function can influence squamous neoplasia at such an early stage, since in mouse multistage SCC development p53 loss appears to influence only the later stages of progression to carcinoma (Kemp *et al*., 1993).

The p53 protein has also been shown to be targeted for degradation by the E6 protein of the more oncogenic human papillomaviruses (HPV; Scheffner *et al*., 1990; Werness *et al*., 1990) and by some investigators to be complexed, but not degraded, by the E6 proteins of the less oncogenic HPV types 6 or 11 (Crook *et al*., 1991). HPV types 2 (de Villiers *et al*., 1985; Adler-Storthz *et al*., 1986), 4 (Yeudall & Campo, 1991), 16 (Maitland *et al*., 1987, 1989; Yeudall & Campo, 1991; Brachman *et al*., 1992), 18 (Yeudall & Campo, 1991) and 33 (Snijders *et al*., 1992) have been reported from malignant SCC-HN, types 6 and 11 from recurrent laryngeal papillomas (Gissman *et al*., 1982; Mounts *et al*., 1982) and types 16 and 18 from papillomas, leukoplakias, dysplasias, keratoses and lichen planus (Loning *et al*., 1985; Maitland *et al*., 1987).

In order to understand further the molecular events which give rise to premalignant head and neck lesions and influence their progression, we have examined the p53 and HPV status of several of these neoplasms and the phenotypically characterised cultures derived from them.

### Materials and methods

### *Tissue collection and pathology*

Tissues, cell cultures and the cell line DOK together with their properties are all listed in Table I. The six adult recurrent papilloma samples (Clark *et al*., 1993*a*), the four erythroplakia cultures (Edington *et al*., 1994) and the cell line DOK (Chang *et al*., 1992) have all been described previously.

Correspondence: E.K. Parkinson.
*The first two authors contributed equally to the study.
+Present address: LCDO, NIDR, NIH, Bethesda, Maryland 20892, USA.
Received 7 January 1994; accepted in revised form 25 April 1994.

*Cultivation and properties of the premalignant keratinocytes*

The human papilloma (BICR P2, BICR P5) and erythroplakia (BICR E1, BICR E2, BICR E4, BICR E5) keratinocytes were cultured on lethally irradiated Swiss 3T3 feeder cells in Dulbecco's modified Eagle medium, 20% (v/v) fetal bovine serum, 0.4 $\mu g\ ml^{-1}$ hydrocortisone and 10 $ng\ ml^{-1}$ cholera toxin as described previously (Edington *et al.*, 1994). DOK cells were cultured in the same way except that 10% (v/v) fetal bovine serum was used and cholera toxin was omitted. The properties of the cells are listed in Table II. The DOK and erythroplakia cultures are known to be composed of transformed keratinocytes since all of these cultures contain low levels of terminally differentiated cells as assessed by cross-linked cornified envelope formation (Table I, Edington *et al.*, 1994). Furthermore, all of these cultures show a low tendency to terminally differentiate when their proliferation is arrested in suspension culture (Edington *et al.*, 1994). Both the papilloma and the erythroplakia cells are diploid, have a limited culture lifespan which is not necessarily longer than normal tongue keratinocytes from adults of the same age group and require essentially the same culture conditions as normal keratinocytes for optimal proliferation. DOK cells possess additional abnormalities in that they are aneuploid, possess an unlimited culture lifespan and have a reduced requirement for serum growth factors and cholera toxin. DOK is therefore phenotypically more abnormal than the other keratinocytes studied.

*Detection of human papilloma virus DNA by polymerase chain reaction (PCR)*

The detection of HPV E6 DNA was performed essentially as described by Burns *et al.* (1993) using SiHa as a positive control for a single copy of HPV 16 DNA per cell and HeLa as a control for HPV 18. SiHa contains only one copy of HPV 16 DNA per cell, and HeLa cells contain 20–100 copies of HPV 18 DNA per cell. However, when the HeLa DNA was diluted 20-fold with normal DNA a signal was still readily detectable, indicating that the HPV 18 detection was sensitive at the level of 1–5 copies of HPV 18 DNA per cell. HPRT primers were used as a control for DNA integrity and PCR efficiency. The primers used to detect the E6 DNA of HPV types 6, 11, 16, 18 and 33 have been described previously (Arends *et al.*, 1989). In some cases PCR products were deposited on nylon filters and probed for HPV sequences as described by Yeudall and Campo (1991). The samples were also screened for the L1 consensus region of HPV types 1, 5, 6, 11, 16, 18, 26, 27, 31, 33, 35, 39, 40, 41, 42, 45, 47, 48, 51, 52, 53, 54, 55, 57 and 59 by the method of Ting and Manos (1990) using the Perkin Elmer Cetus HPV PCR kit and using globin primers to test DNA integrity. These L1 primers also detect at least another 25 types of HPV which are as yet unidentified.

**Table I** Premalignant tissues used in the study

| *Tissue* | *Culture* | *Pathology* | *Malignancy present* |
|---|---|---|---|
| *Papillomas* | | | |
| BICR P1 | No | Papilloma | No |
| BICR P2 | Yes | Papilloma | No |
| BICR P3 | No | Papilloma | No |
| BICR P4 | No | Papilloma | No |
| BICR P5 | Yes | Papilloma | No |
| BICR P6 | No | Papilloma | No |
| *Erythroplakias*[a] | | | |
| BICR E1 | Yes | Carcinoma *in situ* | No |
| BICR E2 | Yes | Carcinoma *in situ* | Yes[b] |
| BICR E3 | No | *Dysplasia* | Yes[c] |
| BICR E4 | Yes | *Carcinoma in situ* | Yes[b] |
| BICR E5 | Yes | Severe dysplasia | Yes[c] |
| *Erythematous leukoplakia*[d] | | | |
| DOK | Yes | Severe dysplasia | Yes[c] |

[a]Data from Edington *et al.* (1994). [b]Malignancy present subsequent to the biopsy being taken. [c]Malignancy present at the time of the biopsy. [d] Data from Chang *et al.* (1992).

*Immunocytochemistry*

Cell cultures and tissue sections were fixed and stained to detect the p53 monoclonal antibodies PAb 240, PAb 1620 (Ball *et al.*, 1984; Milner *et al.*, 1987; Gannon *et al.*, 1990) and PAb 1801 (Banks *et al.*, 1986) exactly as described previously (Burns *et al.*, 1993). Human diploid fibroblasts and human HT29 colon carcinoma cells were used as negative and positive controls respectively. Photographs were taken under bright-field optics using a green filter. Antibodies were obtained from Cambridge Biosciences, Cambridge, UK.

*Direct sequencing of p53*

Direct sequencing of the coding region of the human p53 gene in all samples was accomplished by PCR after reverse transcription of RNA or direct PCR of genomic DNA exactly as described by Burns *et al.* (1993).

### Results

*The status of the p53 tumour-suppressor gene in premalignant tissues and cells*

The p53 coding region was sequenced across exons 5–9 for all six papilloma biopsies and for the cell line DOK since all p53 mutations reported in SCC-HN have so far occurred within this region (Brachman *et al.*, 1992; Jung *et al.*, 1992; Maestro *et al.*, 1992; Sakai & Tsuchida, 1992; Somers *et al.*, 1992; Boyle *et al.*, 1993; Burns *et al.*, 1993; Chung *et al.*, 1993; Nees *et al.*, 1993). The four erythroplakia cultures were sequenced across their entire coding region (Table III). No p53 mutations were found in any of the samples or cultures with the exception of the cell line DOK (Table III), which harboured a 12 bp in-frame deletion of codons 188–191 inclusive (Figure 1). We performed direct sequencing of the reverse-transcribed RNA of DOK, but no expression of the

**Table II** Properties of the keratinocyte cultures and lines used in the study

| *Keratinocytes* | *Ploidy status* | *Abnormal terminal maturation* | *Senescent immortal* | *Reduced growth factor requirements* | *Tumorigenicity* |
|---|---|---|---|---|---|
| *Papillomas* | | | | | |
| BICR P2 | Diploid | ND | Senescent | No | ND |
| BICR P5 | Diploid | ND | Senescent | No | ND |
| *Erythroplakias*[a] | | | | | |
| BICR E1 | ND | Yes | Senescent | No | ND |
| BICR E2 | ND | Yes | Senescent | No | ND |
| BICR E4 | Diploid | Yes | Senescent | No | No |
| BICR E5 | Diploid | Yes | Senescent | No | No |
| *Erythematous leukoplakias*[b] | | | | | |
| DOK | Aneuploid | Yes | Immortal | Yes | No |

[a]Data from Edington *et al.* (submitted). [b]Data from Chang *et al.* (1992). ND, not determined.

**Table III** The p53 status of premalignant keratinocytes and tissues

| *Keratinocytes* | *p53 mutation* | *Codons sequenced* | *HPV present type* | *Analysed in vitro* |
|---|---|---|---|---|
| *Papillomas* | | | | |
| BICR P1 | Normal | 126-331 | HPV 11 | No |
| BICR P2 | Normal | 126-331 | HPV 6 | Yes |
| BICR P3 | Normal | 126-331 | HPV 11 | No |
| BICR P4 | Normal | 126-331 | HPV 6 | No |
| BICR P5 | Normal | 126-331 | HPV 11 | Yes |
| BICR P6 | Normal | 126-331 | HPV 6 | No |
| *Erythroplakias* | | | | |
| BICR E1 | Normal | 1-393 | None | Yes |
| BICR E2 | Normal | 1-393 | None | Yes |
| BICR E3 | ND | ND | None | No |
| BICR E4 | Normal | 1-393 | None | Yes |
| BICR E5 | Normal | 1-393 | None | Yes |
| *Erythroleukoplakia* | | | | |
| DOK | 12 bp deletion of codons 188-191 | 2-96 126-331 | None | Yes |

ND, not determined.

**Figure 1** The 12 bp deletion in DOK cells. The figure shows the sequence of DOK cells aligned with the sequence of another cell line, BICR 56, which is normal in this region (Burns *et al*., 1993). The 12 bp deleted in DOK spanning codons 188-191 is indicated by the bracket on the sequence of BICR 56.

normal p53 allele was detectable, suggesting that the normal allele had been lost or that its expression had been suppressed by some other mechanism.

The 12 bp deletion in the DOK cell line appeared to result in the stabilisation of the p53 protein since it was readily detectable by both PAb 1801 (Figure 2a) and PAb 240 (Figure 2b) antibodies followed by immunoperoxidase staining. Antibody PAb 1620, which does not recognise fixed or mutant p53, gave only weak background staining (Figure 2c),

**Figure 2** Immunostaining of DOK cells with anti-p53 monoclonal antibodies. **a**, PAb 1801. **b**, PAb 240. **c**, PAb 1620. **d**, No antibody (control).

and no staining was seen when the primary antibodies were omitted (Figure 2d). Previously it was reported that DOK cells do not react strongly with PAb 240 and PAb 1801 (Chang *et al*., 1992), but a suboptimal fixation protocol for staining p53 in keratinocytes was employed in the earlier study (see Gusterson *et al*., 1991). The erythroplakia and papilloma cultures all produced a staining pattern with the p53 antibodies which was indistinguishable from normal keratinocytes (data not shown). Also, sections of the erythroplakia biopsy BICR E5 showed no evidence of p53 immunoreactivity when tissue sections of this sample were reacted with antibody PAb 1801 (data not shown).

These results suggest that, with the exception of the cell line DOK none of the premalignant tissues and cultures studied here contain significant numbers of keratinocytes harbouring p53 mutations, since the direct sequencing method is capable of detecting a mutation when only 10-15% of the cells in a sample contain it (Clark *et al*., 1993*b*).

### *Detection of human papillomavirus types in premalignant squamous tissues and cell*

Since several HPV types are known to infect the upper aerodigestive tract and the E6 proteins of some of these types have been proposed to inactivate p53 (Scheffner *et al*., 1990; Werness *et al*., 1990; Crook *et al*., 1991), we screened for the presence of HPV in our samples. Figure 3 shows the detection of the E6 DNA of either HPV types 6 or 11 in each of six adult recurrent human laryngeal papillomas. All the papillomas contained either the E6 DNA of HPV type 6 or 11 but never both (Table III). None of the erythroplakia tissues, their cell cultures or the cell line DOK contained detectable HPV type 6 or 11 E6 DNA (Table III).

### Discussion

It is still unclear how mutation of the p53 tumour-suppressor gene influences the development and progression of SCC-HN. There is evidence that mutations of the p53 gene which lead to stabilisation of the protein give keratinocytes a selective advantage at an early stage of SCC development (Gusterson *et al*., 1991; Dolcetti *et al*., 1992; Ogden *et al*., 1992; Nees *et al*., 1993), and it is possible that these mutants are of the gain-of-function transforming class (Halevy *et al*., 1990). However, experiments using p53 null mice and their heterozygotes suggest that mere loss of p53 function influences only progression from the premalignant to the malignant state during progression to SCC (Kemp *et al*., 1993). Furthermore, some SCC-HN do not possess p53

**Figure 3** Detection of HPV types 6 and 11 in human recurrent papillomas. Lane 1, molecular weight markers; lanes 2–7, HPV 6 primers; lanes 8–13, HPV 11 primers; lane 14, kit control. BICR P1, lanes 2 and 8; BICR P2, lanes 3 and 9; BICR P3, lanes 4 and 10; BICR P4, lanes 5 and 11; BICR P5, lanes 6 and 12; BICR P6, lanes 7 and 13; A = 236 bp HPV fragment. Top arrow = gel top. Bottom arrow = gel bottom.

mutations at all, even at a late stage of tumour progression, and these same tumours lack detectable HPV (Brachman *et al.*, 1992). Therefore, there may be SCCs which arise by a mechanism in which p53 dysfunction cannot bestow a selective advantage on the developing tumour cells until a very late stage, if at all. Therefore, premalignant human oral keratinocyte cultures which lack both p53 mutation and oncogenic HPV types would be useful to test the role of p53 dysfunction in progression towards SCC. In this paper we have tested several human premalignant cultures to identify such cultures.

Six laryngeal papillomas and two of their cell cultures were shown to have normal p53 genes spanning codons 126–331 (Table III), but all of them harboured either HPV 6 or HPV 11 E6 sequences (Figure 3, Table III). Since it has been reported that the E6 protein of HPV types 6 or 11 binds p53 *in vitro* (Crook *et al.*, 1991), it is possible that the presence of these viruses partially inactivates the p53 protein in these cells and may explain the unusually high levels of p53 protein found in the more basal layers of recurrent papillomas *in vivo* which we reported previously (Clark *et al.*, 1993*a*). Therefore, cultures of laryngeal papilloma cells would not be ideal materal to investigate the role of p53 in progression to SCC as they could not be guaranteed to possess a normal-functioning p53 protein.

We also investigated the p53 status of five premalignant keratinocyte cultures which were isolated from either squamous dysplasias or carcinomas *in situ* (Table I). None contained detectable oncogenic HPV E6 or L1 DNA sequences (Table III) so there was no evidence to support the inactivation of p53 by these viruses. The cell line DOK (Chang *et al.*, 1992) did, however, possess a homozygous 12 bp deletion of the p53 coding region (Figure 1 and Table III) and expressed elevated levels of the p53 protein (Figure 2a and b). Since DOK was isolated from dysplastic epithelium adjacent to an SCC of the tongue (Chang *et al.*, 1992) and the presence of p53 mutations has been reported from such lesions (Boyle *et al.*, 1993), it is not surprising that this cell line harbours a p53 mutation. Nevertheless, since DOK is non-tumorigenic (Chang *et al.*, 1992) it should be useful in the study or identification of mutations which cooperate with mutant p53 to effect progression towards SCC.

In contrast, all of the keratinocyte strains derived from premalignant oral erythroplakias lacked a p53 mutation in the coding sequence, and at least two (BICR E4 and BICR E5) have not lost heterozygosity at the p53 locus (Edington *et al.*, 1994), making a mutation outside the coding sequence also unlikely. We have considered several possibilities to explain our results. First, the erythroplakia keratinocytes might be in fact be normal cells since they have diploid karyotypes and senesce in culture (Table II). However, all four BICR keratinocyte strains from erythroplakias showed a reduced rate of terminal maturation when placed in suspension culture (Edington *et al.*, 1994, Table II) and did not proliferate when placed in serum-free MCDB 153 medium (Wille *et al.*, 1984), which supports extensive proliferation of normal keratinocytes (Edington *et al.*, 1994). Second, the erythroplakia cell might be benign, not premalignant; however, this is also unlikely as BICR E5 already contained a developing carcinoma at the time of biopsy and patients BICR E2 and BICR E4 developed SCC within 12 months of the original biopsy date.

It is clear that DOK cells are phenotypically more altered than the erythroplakia cells (Table II) and in addition are aneuploid (Chang *et al.*, 1992) and express high numbers of epidermal growth factor receptors (Stanton *et al.*, 1994). This is not surprising in view of the evidence that one of the functions of p53 is to maintain genetic stability (Bischoff *et al.*, 1990; Kastan *et al.*, 1991; Lane, 1992; Livingstone *et al.*, 1992; Yin *et al.*, 1992) and the data showing that DOK cells possess a homozygous deletion within the p53 gene.

It is, however, still uncertain whether the erythroplakia keratinocytes are at an earlier stage of tumour progression than DOK or whether they represent the precursor lesion of a type of SCC-HN which arises by a p53-independent mechanism. The availability of a series of premalignant erythroplakia keratinocytes which possess normal p53 genes now permits us to address these questions by manipulating the p53 status of these cells.

The authors would like to thank Professor John Wyke for critical reading of the manuscript and the Cancer Research Campaign for financial support.

### References

ADLER-STORTHZ, K., NEWLAND, J.R., TESSIN, B.A., YEUDALL, W.A. & SHILLITOE, E.J. (1986). HPV2 DNA in oral verrucous carcinoma. *J. Oral Pathol.*, **15**, 472–475.

ARENDS, M.J., DONALDSON, Y.K., DUVALL, E., WYLLIE, A.H. & BIRD, C.C. (1991). HPV in full thickness cervical biopsies: high prevalence in CIN 2 and CIN 3 detected by a sensitive PCR method. *J. Pathol.*, **165**, 301–309.

BALL, R.K., SIEGL, B., QUELHORST, S., BRANDER, G. & BRAUN, D.G. (1984). Monoclonal antibodies against simian virus 40 nuclear large T tumour antigen: epitope mapping, papova virus cross-reaction and cell surface staining. *EMBO J.*, **3**, 1485-1491.

BANKS, L., MATLASHEWSKI, G. & CRAWFORD, L. (1986). Isolation of human-p53-specific monoclonal antibodies and their use in the studies of human p53 expression. *Eur. J. Biochem.*, **259**, 529–534.

BISCHOFF, F.Z., YIM, S.O., PATHAK, S., GRANT, G., SICILIANO, M.J., GIOVANELLA, B.C., STRONG, L.C. & TAINSKY, M.A. (1990). Spontaneous abnormalities in normal fibroblasts from patients with Li-Fraumeni cancer syndrome: aneuploidy and immortalization. *Cancer Res.*, **50**, 7979–7984.

BOYLE, J.O., HAKIM, J., KOCH, W., VAN DER RIET, P., HRUBAN, R.H., ROA, R.A., CORREO, R., ELBY, Y.J., RUPPERT, J.M. & SIDRANSKY, D. (1993). The incidence of p53 mutations increases with progression of head and neck cancer. *Cancer Res.*, **53**, 4477–4480.

BRACHMAN, D.G., GRAVES, D., VOKES, E., BECKETT, M., HARAF, D., MONTAG, A., DUNPHY, E., MICK, R., YANDELL, D. & WEICHSELBAUM, R.R. (1992). Occurrence of p53 gene deletions and human papilloma virus infection in human head and neck cancer. *Cancer Res.*, **52**, 4832–4836.

BURNS, J.E., BAIRD, M.C., CLARK, L.J., BURNS, P.A., EDINGTON, K., CHAPMAN, C., MITCHELL, R., ROBERTSON, G., SOUTAR, D. & PARKINSON, E.K. (1993). Gene mutations and increased levels of p53 protein in human squamous cell carcinomas and their cell lines. *Br. J. Cancer*, **67**, 1274-1284.

CHANG, S.E., FOSTER, S., BETTS, D. & MARNOCK, W.E. (1992). DOK, a cell line established from human dysplastic oral mucosa, shows a partially transformed non-malignant phenotype. *Int. J. Cancer*, **52**, 896-902.

CHUNG, K.Y., MUKHOPADHYAY, T., KIM, J., CASSON, A., RO, J.Y., GOEPFERT, H., HONG, W.K. & ROTH, J.A. (1993). Discordant p53 gene mutations in primary head and neck cancers and corresponding second primary cancers of the upper aerodigestive tract. *Cancer Res.*, **53**, 1676-1683.

CLARK, L.J., MACKENZIE, K. & PARKINSON, E.K. (1993*a*). Elevated levels of the p53 tumour suppressor protein in the basal layer of recurrent laryngeal papillomas. *Clin. Otolaryngol.*, **18**, 63-65.

CLARK, L.J., EDINGTON, K., SWAN, I.R.C., McLAY, K.A., NEWLANDS, W.J., WILLS, L.C., YOUNG, H.A., JOHNSTON, P.W., MITCHELL, R., ROBERTSON, G., SOUTAR, D., PARKINSON, E.K. & BIRNIE, G.D. (1993*b*). The absence of Harvey *ras* mutations during development and progression of squamous cell carcinomas of the head and neck. *Br. J. Cancer*, **68**, 617-620.

CROOK, T., TIDY, J.A. & VOUSDEN, K.H. (1991). Degradation of p53 can be targeted by HPV E6 sequences distinct from those required for p53 binding and transactivation. *Cell*, **67**, 547-556.

DE VILLIERS, E.M., WEIDAUER, H., OTTO, H. & ZUR HAUSEN, H. (1985). Papilloma DNA in human tongue carcinomas. *Int. J. Cancer*, **36**, 575-578.

DOLCETTI, R., DOGLIONI, C., MAESTRO, R., GASPAROTTO, D., BARZAN, L., PASTORE, A., ROMANELLI, M. & BOIOCCHI, M. (1992). p53 overexpression is an early event in the development of human squamous cell carcinoma of the larynx - genetic and prognostic implications. *Int. J. Cancer*, **52**, 178-182.

EDINGTON, K.G., BERRY, I.J., O'PREY, M., BURNS, J.E., CLARK, L.J., MITCHELL, R., ROBERTSON, G., SOUTAR, D., COGGINS, L.W. & PARKINSON, E.K. (1994). Cultivation and phenotypic characterisation of premalignant oral erythroplakia and malignant squamous cell carcinoma cells at different stages of tumour progression. In *Culture of Tumor Cells, Culture of Specialized Cells*. Freshney, R.I. (ed.). Wiley-Liss: New York (in press).

FIELD, J.K., SPANDIDOS, D.A., MALLIRI, A., GOSNEY, J.R., YIAGNISIS, M. & STELL, P.M. (1991). Elevated p53 expression correlates with a history of heavy smoking in squamous cell carcinoma of the head and neck. *Br. J. Cancer*, **64**, 573-577.

GANNON, J.V., GREAVES, R., IGGO, R. & LANE, D.P. (1990). Activating mutants in p53 produce common conformational effects. A monoclonal antibody specific for the mutant form. *EMBO J.*, **9**, 1591-1602.

GISSMAN, L., DIEHL, V., SCHULTZ-COULON, H.-J. & ZUR HAUSEN, H. (1982). Molecular cloning and characterisation of human papilloma virus DNA derived from a laryngeal papilloma. *J. Virol.*, **44**, 393-400.

GUSTERSON, B.A., ANBAZHAGEN, R., WARREN, W., MIDGELY, C., LANE, D.P., O'HARE, M., STAMPS, A., CARTER, R. & JAYATILAKE, H. (1991). Expression of p53 in premalignant and malignant squamous epithelium. *Oncogene*, **6**, 1785-1789.

HALEVY, O., MICHAELOVITCH, D. & OREN, M. (1990). Different tumor-derived p53 mutants exhibit distinct biological activities. *Science*, **250**, 113-116.

JUNG, M., NOTARIO, V. & DRITCHILO, A. (1992). Mutations in the p53 gene in radiation-sensitive and -resistant human squamous carcinoma cells *Cancer Res.*, **52**, 6390-6393.

KASTAN, M.B., ONYEKWERE, O., SIDRANSKY, D., VOGELSTEIN, B. & CRAIG, R.W. (1991). Participation of p53 protein in the cellular response to DNA damage. *Cancer Res.*, **51**, 6304-6311.

KEMP, C.J., DONEHOWER, L.A., BRADLEY, A. & BALMAIN, A. (1993). Reduction of p53 gene dosage does not increase initiation or promotion but enhances malignant progression of chemically induced skin tumours. *Cell*, **74**, 813-822.

LANE, D.P. (1992). p53, guardian of the genome. *Nature*, **358**, 15-16.

LIVINGSTONE, L.R., WHITE, A., SPROUSE, J., LIVANOS, E., JACKS, T. & TLSTY, T. (1992). 'Altered cell cycle arrest and gene amplification potential accompany loss of wild-type p53. *Cell*, **70**, 923-935.

LONING, T., IKENBERG, H., BECKER, J., GISSMAN, L., HOEPFNER, I. & ZUR HAUSEN, H. (1985). Analysis of oral papillomas, leukoplakias and invasive carcinomas for human papillomavirus type related DNA. *J. Invest. Dermatol.*, **88**, 417-420.

MAESTRO, R., DOLCETTI, R., GASPAROTTO, C., DOGLIONI, C., PELUCCHI, S., BARZAN, L., GRANDI, E. & BOIOCCHI, M. (1992). High frequency of p53 gene alterations associated with protein overexpression in human squamous cell carcinoma of the larynx. *Oncogene*, **7**, 1159-1166.

MAITLAND, N.J., COX, M.F., LYNAS, C., PRIME, S.S. & SCULLY, C. (1987). Detection of human papillomavirus DNA in biopsies of human oral tissue. *Br. J. Cancer*, **56**, 245-250.

MAITLAND, N.J., BROMIDGE, T., COX, M.F., CRANE, I.J., PRIME, S.S. & SCULLY, C. (1989). Detection of human papillomavirus genes in human oral tissue biopsies and cultures by polymerase chain reaction. *Br. J. Cancer*, **59**, 698-703.

MILLION, R.R., CASSISI, N.J. & CLARK, J.R. (1989). Cancer of the head and neck. In *Cancer: Principles and Practice of Oncology*, De Vitae Jr, V.T., Hellman, T. & Rosenberg, S.A. (eds) pp. 488-590. J.B. Lippincott: Philadelphia.

MILNER, J., COOK, A. & SHELDON, M. (1987). A new anti-p53 monoclonal antibody, previously reported to be directed against the large T antigen of simian virus 40. *Oncogene*, **1**, 453-455.

MOUNTS, P., SHAH, K.V. & KASHIMA, H. (1982). Viral etiology of juvenile- and adult-onset squamous papilloma of the larynx. *Proc. Natl Acad. Sci. USA.*, **79**, 5425-5429.

NEES, M., HOMANN, N., DISCHER, H., ANDL, T., ENDERS, C., HEROLD-MENDE, C., SCHUMANN, A. & BOSCH, F.X. (1993). Expression of mutated p53 occurs in tumor-distant epithelia of head and neck cancer patients: a possible molecular basis for the development of multiple tumors. *Cancer Res.*, **53**, 4189-4196.

OGDEN, G.R., KIDDIE, R.A., LUNNY, D.P. & LANE, D.P. (1992). Assessment of p53 protein expression in normal, benign and malignant oral mucosa. *J. Pathol.*, **166**, 389-394.

OREN, M., MALTZMAN, W. & LEVINE, A.J. (1981). Post-translational regulation of the 54k cellular antigen in normal and transformed cells. *Mol. Cell Biol.*, **1**, 101-110.

PINDBORG, J.J. (1984). Control of oral cancer in developing countries. *Bull. WHO*, **62**, 817-830.

PINDBORG, J.J. (1985). Oral precancer. In *Surgical Pathology of the Head and Neck*, Vol. 1, Barnes, I. (ed.) pp. 279-331. Marcel Dekker: New York.

SAKAI, E. & TSUCHIDA, N. (1992). Most human squamous cell carcinomas in the oral cavity contain mutated p53 tumor-suppressor genes. *Oncogene*, **7**, 927-933.

SCHEFFNER, M., WERNESS, B.A., HUMBREGTSE, J.M., LEVINE, A.J. & HOWLEY, P.M. (1990). The E6 oncoprotein encoded by human papillomaviruses 16 and 18 promotes the degradation of p53. *Cell*, **63**, 1129-1136.

SNIJDERS, P.J.F., CROMME, F.V., VAN DEN BRULE, A.J.C., SCHRIJNEMAKERS, H.F.J., SNOW, G.B., MEIJER, C.J.L.M. & WALBOOMERS, J.M.M. (1992). Prevalence and expression of human papillomavirus in tonsillar carcinomas, indicating a possible viral etiology. *Int. J. Cancer*, **51**, 845-850.

SOMERS, K.D., MERRICK, M.A., LOPEZ, M.E., INCOGNITO, L.S., SCHECHTER, G.L. & CASEY, G. (1992). Frequent p53 mutations in head and neck cancer. *Cancer Res.*, **52**, 5997-6000.

STANTON, P., RICHARDS, S., REEVES, J., NIKOLIC, M., EDINGTON, K., CLARK, L., ROBERTSON, G., SOUTAR, D., HENDLER, F.J., COOKE, T., PARKINSON, E.K. & OZANNE, B. (1994). EGF receptor expression by human squamous cell carcinomas of the head and neck tumours, cell lines and xenografts. *Br. J. Cancer*, **70**, 427-433.

TING, Y. & MANOS, M.M. (1990). In *PCR Protocols: A Guide to Methods and Applications*, Innes, M.A., Gelfand, D.H., Sninsky, J.J. & White, T.J. (eds) pp. 356-367. Academic Press: San Diego, CA.

WERNESS, B.A., LEVINE, A.J. & HOWLEY, P.M. (1990). Association of human papillomavirus type 16 and 18 E6 proteins with p53. *Science*, **248**, 76-79.

WILLE, J.J., PITTELKOW, M.R., SHIPLEY, G.D. & SCOTT, R.E. (1984). Integrated control of growth and differentiation of normal prokeratinocytes cultured in serum-free medium: clonal analyses, growth kinetics and cell cycle studies. *J. Cell Physiol.*, **121**, 31-44.

YEUDALL, W.A. & CAMPO, M.S. (1991). Human papillomavirus DNA in biopsies of oral tissues. *J. Gen. Virol.*, **72**, 173-176.

YIN, Y., TAINSKY, M.A., BISCHOFF, F.Z., STRONG, L.C. & WAHL, G.M. (1992). Wild-type p53 restores cell cycle control and inhibits gene amplification in cells with mutant p53 alleles. *Cell*, **70**, 937-948.


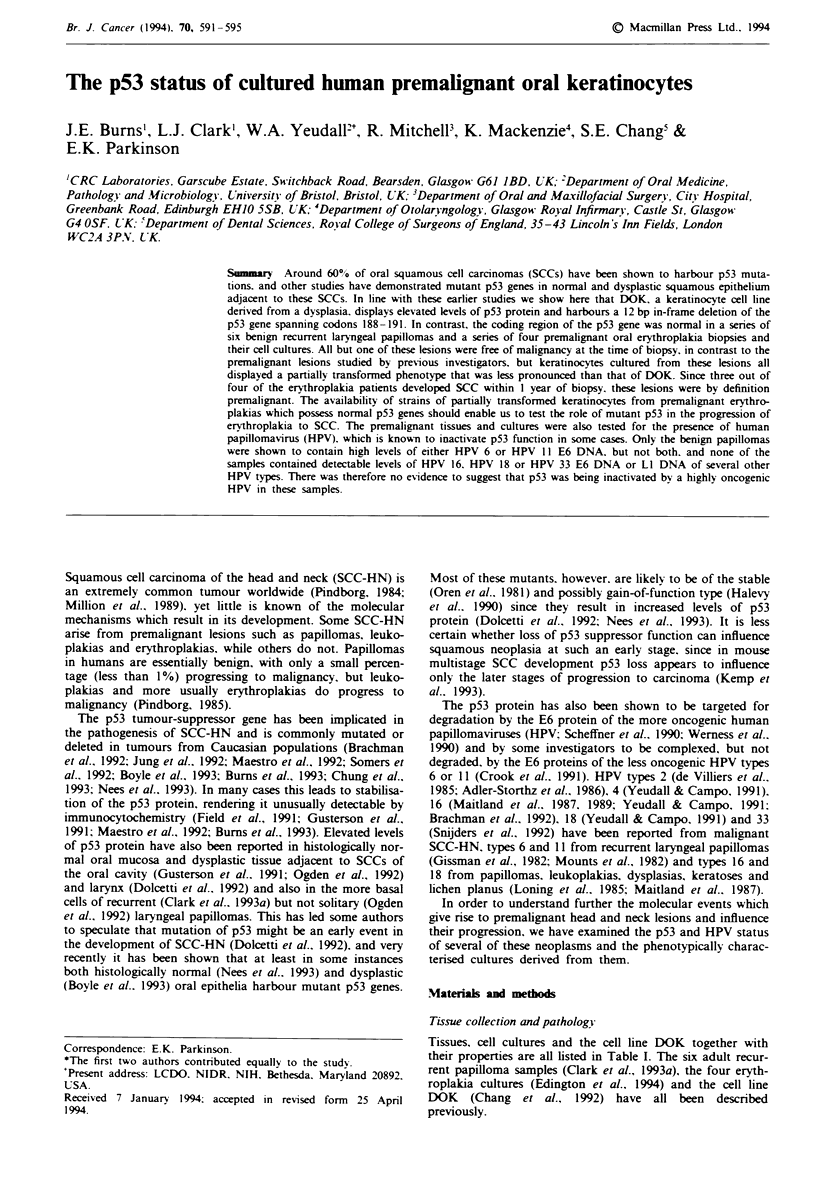

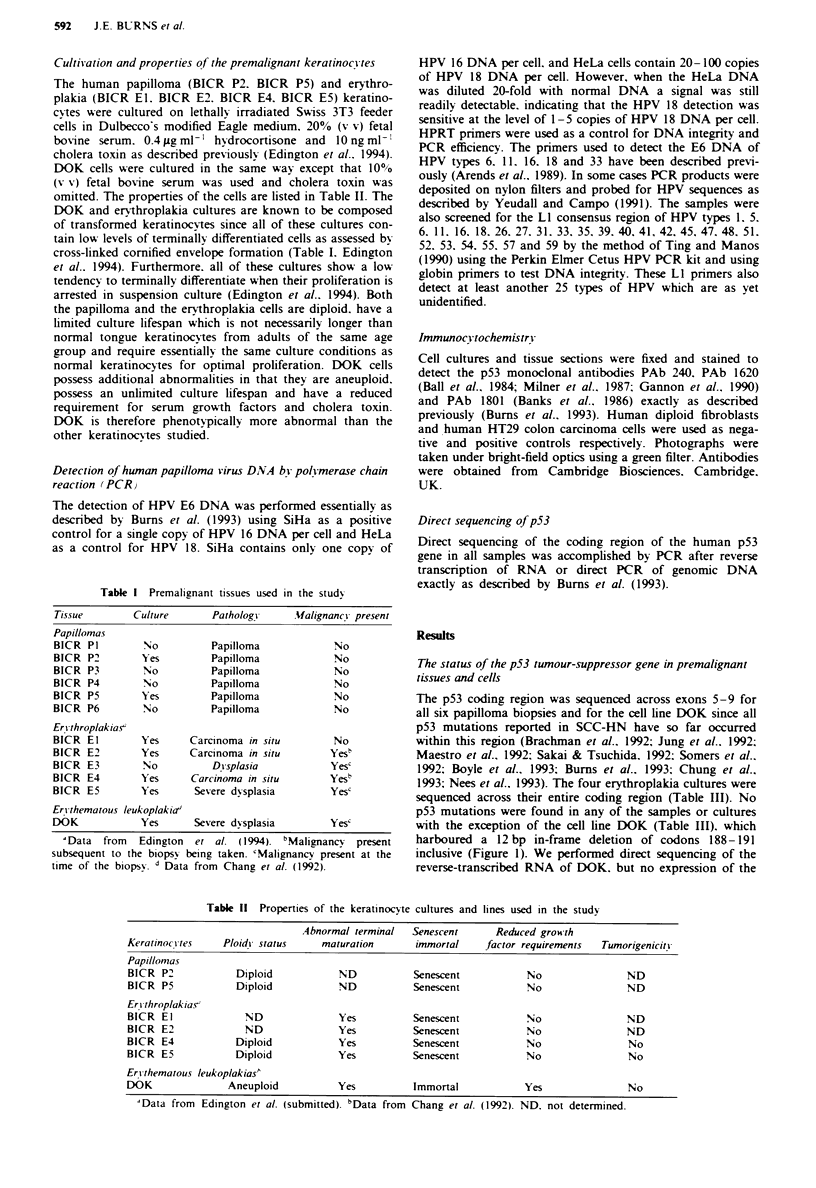

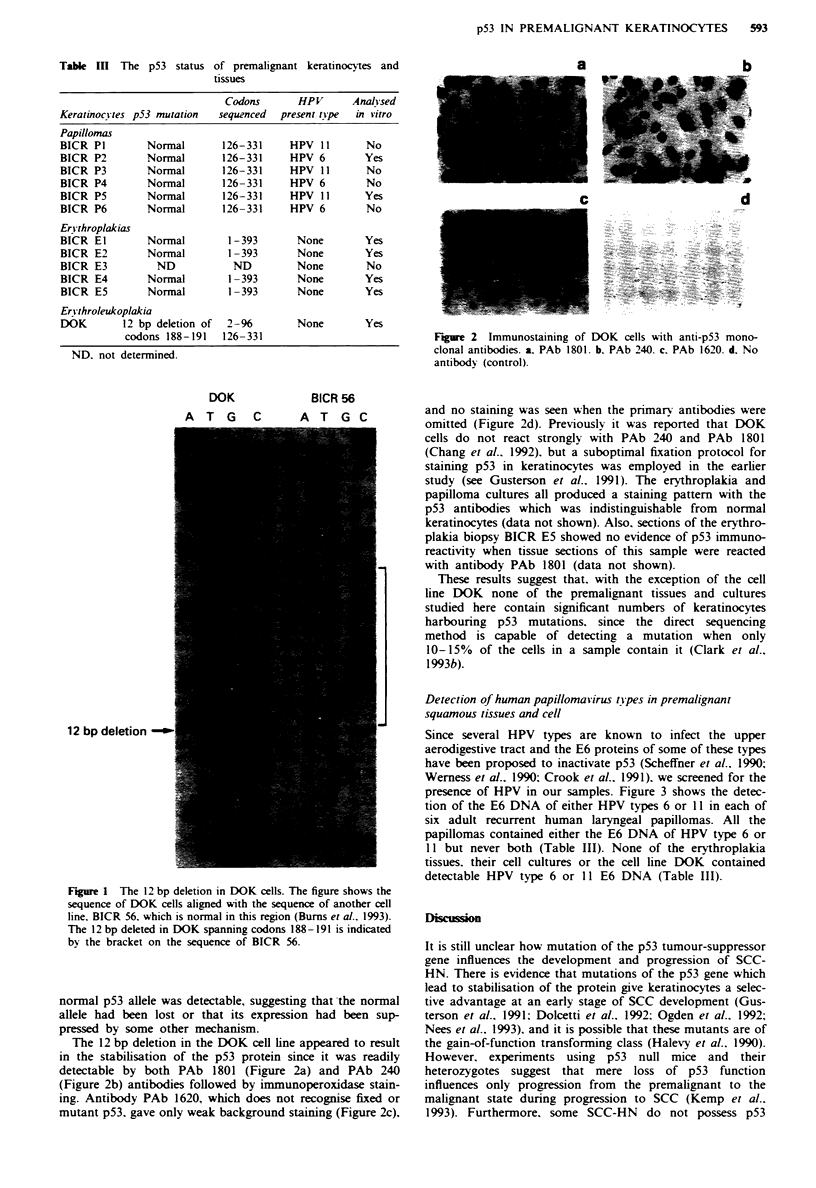

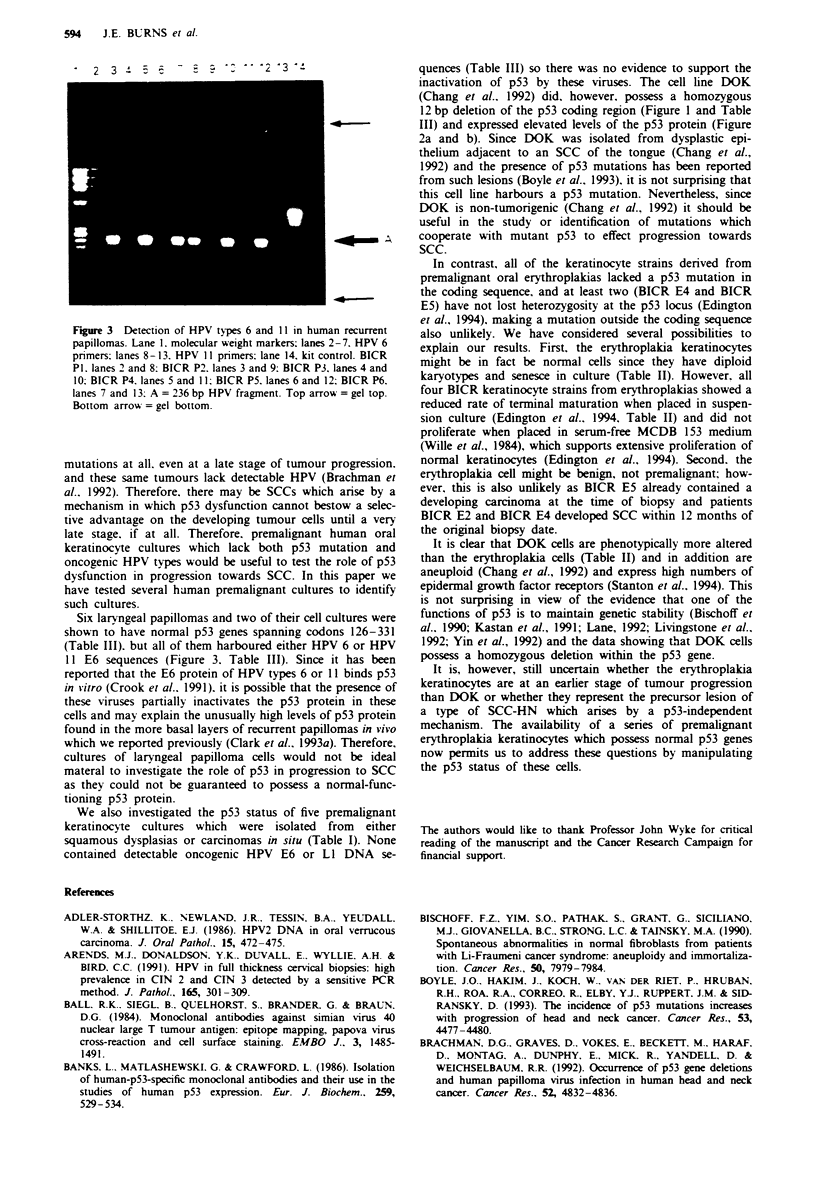

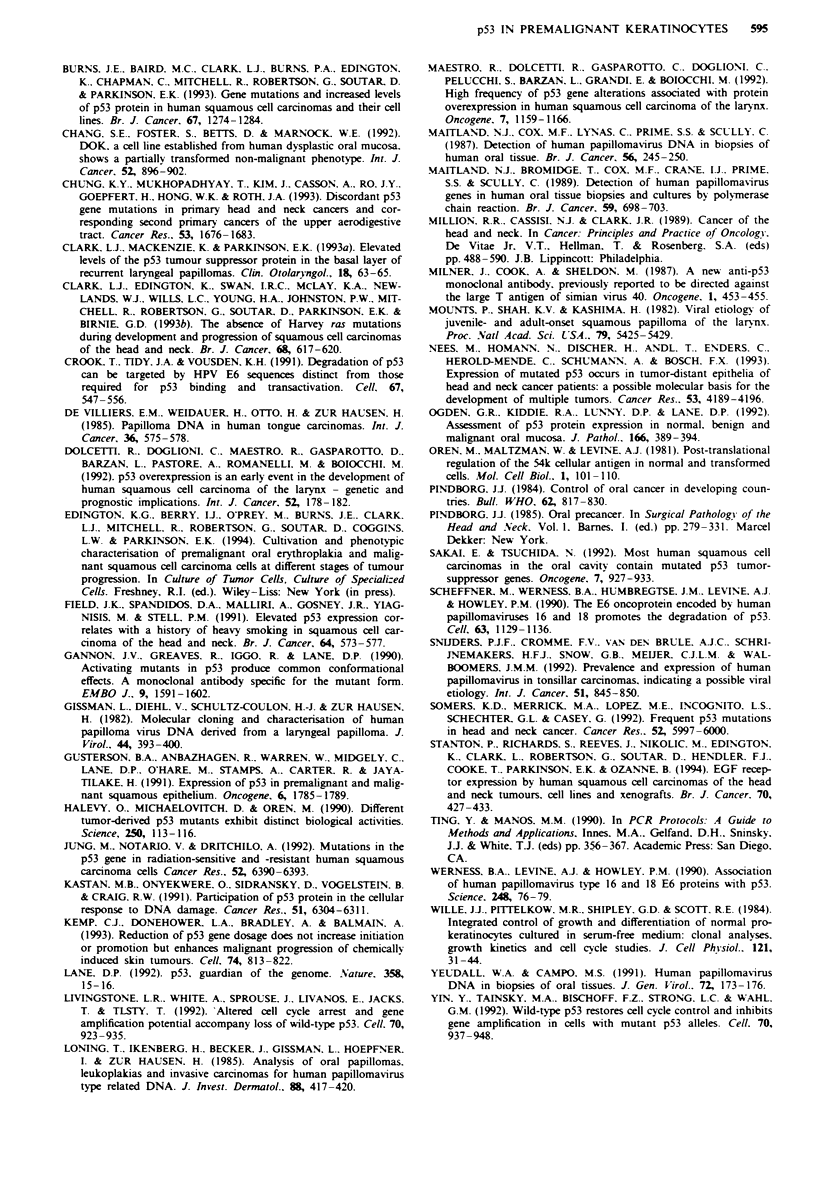


## References

[OCR_00500] Adler-Storthz K., Newland J. R., Tessin B. A., Yeudall W. A., Shillitoe E. J. (1986). Human papillomavirus type 2 DNA in oral verrucous carcinoma.. J Oral Pathol.

[OCR_00506] Arends M. J., Donaldson Y. K., Duvall E., Wyllie A. H., Bird C. C. (1991). HPV in full thickness cervical biopsies: high prevalence in CIN 2 and CIN 3 detected by a sensitive PCR method.. J Pathol.

[OCR_00511] Ball R. K., Siegl B., Quellhorst S., Brandner G., Braun D. G. (1984). Monoclonal antibodies against simian virus 40 nuclear large T tumour antigen: epitope mapping, papova virus cross-reaction and cell surface staining.. EMBO J.

[OCR_00518] Banks L., Matlashewski G., Crawford L. (1986). Isolation of human-p53-specific monoclonal antibodies and their use in the studies of human p53 expression.. Eur J Biochem.

[OCR_00522] Bischoff F. Z., Yim S. O., Pathak S., Grant G., Siciliano M. J., Giovanella B. C., Strong L. C., Tainsky M. A. (1990). Spontaneous abnormalities in normal fibroblasts from patients with Li-Fraumeni cancer syndrome: aneuploidy and immortalization.. Cancer Res.

[OCR_00532] Boyle J. O., Hakim J., Koch W., van der Riet P., Hruban R. H., Roa R. A., Correo R., Eby Y. J., Ruppert J. M., Sidransky D. (1993). The incidence of p53 mutations increases with progression of head and neck cancer.. Cancer Res.

[OCR_00539] Brachman D. G., Graves D., Vokes E., Beckett M., Haraf D., Montag A., Dunphy E., Mick R., Yandell D., Weichselbaum R. R. (1992). Occurrence of p53 gene deletions and human papilloma virus infection in human head and neck cancer.. Cancer Res.

[OCR_00549] Burns J. E., Baird M. C., Clark L. J., Burns P. A., Edington K., Chapman C., Mitchell R., Robertson G., Soutar D., Parkinson E. K. (1993). Gene mutations and increased levels of p53 protein in human squamous cell carcinomas and their cell lines.. Br J Cancer.

[OCR_00552] Chang S. E., Foster S., Betts D., Marnock W. E. (1992). DOK, a cell line established from human dysplastic oral mucosa, shows a partially transformed non-malignant phenotype.. Int J Cancer.

[OCR_00558] Chung K. Y., Mukhopadhyay T., Kim J., Casson A., Ro J. Y., Goepfert H., Hong W. K., Roth J. A. (1993). Discordant p53 gene mutations in primary head and neck cancers and corresponding second primary cancers of the upper aerodigestive tract.. Cancer Res.

[OCR_00573] Clark L. J., Edington K., Swan I. R., McLay K. A., Newlands W. J., Wills L. C., Young H. A., Johnston P. W., Mitchell R., Robertson G. (1993). The absence of Harvey ras mutations during development and progression of squamous cell carcinomas of the head and neck.. Br J Cancer.

[OCR_00565] Clark L. J., MacKenzie K., Parkinson E. K. (1993). Elevated levels of the p53 tumour suppressor protein in the basal layer of recurrent laryngeal papillomas.. Clin Otolaryngol Allied Sci.

[OCR_00578] Crook T., Tidy J. A., Vousden K. H. (1991). Degradation of p53 can be targeted by HPV E6 sequences distinct from those required for p53 binding and trans-activation.. Cell.

[OCR_00589] Dolcetti R., Doglioni C., Maestro R., Gasparotto D., Barzan L., Pastore A., Romanelli M., Boiocchi M. (1992). p53 over-expression is an early event in the development of human squamous-cell carcinoma of the larynx: genetic and prognostic implications.. Int J Cancer.

[OCR_00607] Field J. K., Spandidos D. A., Malliri A., Gosney J. R., Yiagnisis M., Stell P. M. (1991). Elevated P53 expression correlates with a history of heavy smoking in squamous cell carcinoma of the head and neck.. Br J Cancer.

[OCR_00611] Gannon J. V., Greaves R., Iggo R., Lane D. P. (1990). Activating mutations in p53 produce a common conformational effect. A monoclonal antibody specific for the mutant form.. EMBO J.

[OCR_00617] Gissmann L., Diehl V., Schultz-Coulon H. J., zur Hausen H. (1982). Molecular cloning and characterization of human papilloma virus DNA derived from a laryngeal papilloma.. J Virol.

[OCR_00626] Gusterson B. A., Anbazhagan R., Warren W., Midgely C., Lane D. P., O'Hare M., Stamps A., Carter R., Jayatilake H. (1991). Expression of p53 in premalignant and malignant squamous epithelium.. Oncogene.

[OCR_00629] Halevy O., Michalovitz D., Oren M. (1990). Different tumor-derived p53 mutants exhibit distinct biological activities.. Science.

[OCR_00634] Jung M., Notario V., Dritschilo A. (1992). Mutations in the p53 gene in radiation-sensitive and -resistant human squamous carcinoma cells.. Cancer Res.

[OCR_00642] Kastan M. B., Onyekwere O., Sidransky D., Vogelstein B., Craig R. W. (1991). Participation of p53 protein in the cellular response to DNA damage.. Cancer Res.

[OCR_00644] Kemp C. J., Donehower L. A., Bradley A., Balmain A. (1993). Reduction of p53 gene dosage does not increase initiation or promotion but enhances malignant progression of chemically induced skin tumors.. Cell.

[OCR_00650] Lane D. P. (1992). Cancer. p53, guardian of the genome.. Nature.

[OCR_00654] Livingstone L. R., White A., Sprouse J., Livanos E., Jacks T., Tlsty T. D. (1992). Altered cell cycle arrest and gene amplification potential accompany loss of wild-type p53.. Cell.

[OCR_00660] Löning T., Ikenberg H., Becker J., Gissmann L., Hoepfer I., zur Hausen H. (1985). Analysis of oral papillomas, leukoplakias, and invasive carcinomas for human papillomavirus type related DNA.. J Invest Dermatol.

[OCR_00666] Maestro R., Dolcetti R., Gasparotto D., Doglioni C., Pelucchi S., Barzan L., Grandi E., Boiocchi M. (1992). High frequency of p53 gene alterations associated with protein overexpression in human squamous cell carcinoma of the larynx.. Oncogene.

[OCR_00680] Maitland N. J., Bromidge T., Cox M. F., Crane I. J., Prime S. S., Scully C. (1989). Detection of human papillomavirus genes in human oral tissue biopsies and cultures by polymerase chain reaction.. Br J Cancer.

[OCR_00675] Maitland N. J., Cox M. F., Lynas C., Prime S. S., Meanwell C. A., Scully C. (1987). Detection of human papillomavirus DNA in biopsies of human oral tissue.. Br J Cancer.

[OCR_00690] Milner J., Cook A., Sheldon M. (1987). A new anti-p53 monoclonal antibody, previously reported to be directed against the large T antigen of simian virus 40.. Oncogene.

[OCR_00696] Mounts P., Shah K. V., Kashima H. (1982). Viral etiology of juvenile- and adult-onset squamous papilloma of the larynx.. Proc Natl Acad Sci U S A.

[OCR_00702] Nees M., Homann N., Discher H., Andl T., Enders C., Herold-Mende C., Schuhmann A., Bosch F. X. (1993). Expression of mutated p53 occurs in tumor-distant epithelia of head and neck cancer patients: a possible molecular basis for the development of multiple tumors.. Cancer Res.

[OCR_00706] Ogden G. R., Kiddie R. A., Lunny D. P., Lane D. P. (1992). Assessment of p53 protein expression in normal, benign, and malignant oral mucosa.. J Pathol.

[OCR_00711] Oren M., Maltzman W., Levine A. J. (1981). Post-translational regulation of the 54K cellular tumor antigen in normal and transformed cells.. Mol Cell Biol.

[OCR_00725] Sakai E., Tsuchida N. (1992). Most human squamous cell carcinomas in the oral cavity contain mutated p53 tumor-suppressor genes.. Oncogene.

[OCR_00732] Scheffner M., Werness B. A., Huibregtse J. M., Levine A. J., Howley P. M. (1990). The E6 oncoprotein encoded by human papillomavirus types 16 and 18 promotes the degradation of p53.. Cell.

[OCR_00736] Snijders P. J., Cromme F. V., van den Brule A. J., Schrijnemakers H. F., Snow G. B., Meijer C. J., Walboomers J. M. (1992). Prevalence and expression of human papillomavirus in tonsillar carcinomas, indicating a possible viral etiology.. Int J Cancer.

[OCR_00743] Somers K. D., Merrick M. A., Lopez M. E., Incognito L. S., Schechter G. L., Casey G. (1992). Frequent p53 mutations in head and neck cancer.. Cancer Res.

[OCR_00751] Stanton P., Richards S., Reeves J., Nikolic M., Edington K., Clark L., Robertson G., Souter D., Mitchell R., Hendler F. J. (1994). Epidermal growth factor receptor expression by human squamous cell carcinomas of the head and neck, cell lines and xenografts.. Br J Cancer.

[OCR_00762] Werness B. A., Levine A. J., Howley P. M. (1990). Association of human papillomavirus types 16 and 18 E6 proteins with p53.. Science.

[OCR_00769] Wille J. J., Pittelkow M. R., Shipley G. D., Scott R. E. (1984). Integrated control of growth and differentiation of normal human prokeratinocytes cultured in serum-free medium: clonal analyses, growth kinetics, and cell cycle studies.. J Cell Physiol.

[OCR_00774] Yeudall W. A., Campo M. S. (1991). Human papillomavirus DNA in biopsies of oral tissues.. J Gen Virol.

[OCR_00778] Yin Y., Tainsky M. A., Bischoff F. Z., Strong L. C., Wahl G. M. (1992). Wild-type p53 restores cell cycle control and inhibits gene amplification in cells with mutant p53 alleles.. Cell.

[OCR_00584] de Villiers E. M., Weidauer H., Otto H., zur Hausen H. (1985). Papillomavirus DNA in human tongue carcinomas.. Int J Cancer.

